# Analysis of Microstructure and Pore Formation Mechanism in Magnetic Pulse-Assisted Semi-Solid Brazed Joints of Cu/Al Tubes

**DOI:** 10.3390/ma18225121

**Published:** 2025-11-11

**Authors:** Zhenglei Rui, Shangyu Huang, Huajun Wang

**Affiliations:** School of Materials Science and Engineering, Wuhan University of Technology, Wuhan 430070, China

**Keywords:** MPASSB, Cu/Al tubes, element diffusion, interface interaction, pores

## Abstract

In this study, the joining of Cu/Al tubes was achieved using the magnetic pulse-assisted semi-solid brazing (MPASSB) technique. A coupled finite element method–smoothed particle hydrodynamics (FEM-SPH) model was established to analyze the influence mechanism of solid–liquid interface interaction on pore formation during the brazing forming process. The results indicate that the MPASSB technique can produce Cu/Al tube joints with excellent metallurgical bonding and performance at 390 °C, and no brittle Cu/Al intermetallic compounds (IMCs) are formed in the joints. Additionally, a stronger solid–liquid interface interaction and a higher surface roughness of the tubes lead to easier peeling of the copper matrix from the interface, thereby promoting pore formation. Mechanical property tests show that the shear strength of the joints prepared by this method can reach 63.3 MPa, and the fracture occurs in the brazing seam area adjacent to the copper–side interface. The MPASSB technique is expected to provide a feasible technical approach for the high-quality joining of dissimilar Cu/Al materials.

## 1. Introduction

In the joining of Cu and Al, two core bottlenecks limit joint quality and service performance: the presence of oxide layers on base metal surfaces and the formation of brittle layered IMCs at the interface [1,2]. Additionally, Cu and Al exhibit significant differences in key physical properties—including melting point, thermal expansion coefficient, and thermal conductivity. These disparities induce substantial residual stress and strain in welded joints, which further elevates the risk of crack initiation in brittle IMCs during service and severely impairs the reliability of joined structures. Currently, the most prevalent Cu/Al joining technologies in academia and industry include friction stir welding (FSW), magnetic pulse welding (MPW), laser welding, and brazing.

Conventional FSW employs a stirring tool to drive base metals into severe plastic deformation. This process effectively removes surface oxide layers and then achieves interfacial metallurgical bonding via frictional heat [3]. However, it frequently generates brittle IMCs (e.g., Al_4_Cu_9_ and Al_2_Cu) at the Cu/Al interface, which degrade the joint’s mechanical properties. To address this issue, Hou [4] proposed an improved approach: inserting a nickel (Ni) interlayer between Cu and Al plates. By prioritizing reactions between Ni and Cu/Al to inhibit brittle phase formation, the joint’s mechanical properties were significantly enhanced. In contrast, MPW relies on electromagnetic forces to drive base metals into impact, generating high-speed metal jets. While removing interfacial oxide layers, MPW leverages instantaneous impact-induced high temperatures to achieve metallurgical bonding [5,6]. Nevertheless, this technology fails to suppress brittle IMC formation, and joints are prone to crack defects—restricting its application in high-reliability scenarios [7].

Among fusion welding techniques, laser welding is currently one of the most suitable for Cu/Al joining. Its low heat input and high welding speed not only minimize the heat-affected zone (HAZ) but also effectively prevent excessive growth of brittle IMCs, while reducing residual stress and strain in joints. For instance, Yan [8] adjusted laser power to reduce the width of layered brittle IMCs in Cu/Al laser-welded joints to 6 μm, markedly improving joint performance.

Brazing represents another widely used Cu/Al joining technology, with the ability to inhibit brittle IMC formation by optimizing the brazing temperature, holding time, or selecting novel filler metals. Among brazing methods, ultrasonic brazing [9] performs particularly well: it utilizes the ultrasonic cavitation effect to efficiently remove oxide layers on base metal surfaces within seconds, enabling interfacial metallurgical bonding while effectively suppressing brittle IMC formation.

For Cu/Al tube joining, MPW and flame brazing remain mainstream technologies. However, neither can effectively resolve the issue of interfacial brittle IMCs, making it challenging to meet high-reliability joining requirements. In response, Huang et al. [10] proposed the MPASSB method. This technique uses electromagnetic forces to drive Al tubes into extrusion of filler metal, facilitating both high-speed shear rheology of semi-solid filler metal and their full interaction with base metals. Ultimately, this achieves oxide film removal and interfacial metallurgical bonding. Nevertheless, the existing MPASSB process still exhibits room for optimization and urgently requires further research and refinement.

Building on the above research status, this study was focused on Cu/Al tubes joining and conducts joining experiments using the MPASSB method. It focuses on analyzing the microstructural characteristics of the joints and, based on the FEM-SPH approach, explores in depth the formation mechanism of pores in the joints under solid–liquid coupling interaction. The objective is to provide theoretical support for optimizing the MPASSB process and realizing high-quality Cu/Al tubes joining.

## 2. Materials and Method

### 2.1. Materials

The 1060-O aluminum tubes were used, with an outer diameter of 19 mm and a wall thickness of 1 mm. The T2-copper tubes featured an outer diameter of 15 mm and a wall thickness of 1.5 mm. The filler metal adopted was a Zn-15Al alloy, which was processed via rolling to achieve a thickness of 400 μm and an axial length of 8 mm. Among these materials, the Cu and Al tubes were procured from Shanghai Pengsheng Metal Materials Co., Ltd. (Shanghai, China), whereas the Zn-15Al filler metal was prepared through laboratory-scale smelting. The chemical compositions of the copper tube, aluminum tube, and filler metal are provided in the authors’ previous studies [11]. Additionally, differential scanning calorimetry (DSC, TA-DSC2500), manufactured by Shanghai Luwen Scientific Instruments Co., Ltd. (Shanghai, China), was conducted to determine the solid–liquid phase transition temperatures of the Zn-15Al filler metal. As shown in the previous results [12], this filler metal exhibits a solidus temperature of 382.4 °C and a liquidus temperature of 459.9 °C.

### 2.2. Experimental Methods

**Figure 1:**
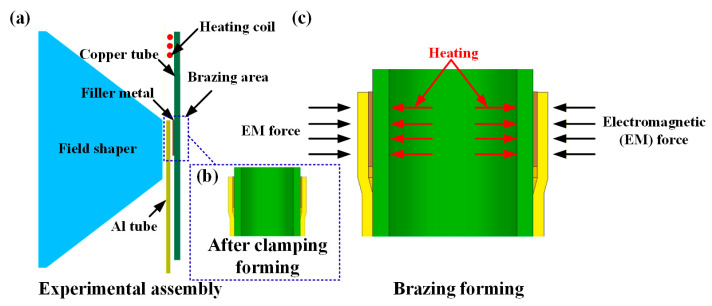
Schematic diagrams of magnetic pulse-assisted semi-solid brazing experiment. (**a**) Schematic of clamping assembly; (**b**) schematic of joint after clamping; (**c**) schematic of brazing forming.

The MPASSB method consists of two stages: clamping forming and brazing forming. Before the experiment, the coil, field shaper, heating coil, Cu/Al tubes, and filler metal were clamped according to the configuration shown in Figure 1a. During the clamping forming stage, the capacitor was charged to 5000 V and then discharged. The Cu/Al tubes and filler metal were clamped tightly by the electromagnetic force generated, as illustrated in Figure 1b. For the brazing forming stage, the capacitor was first recharged to the set voltage (5000 V and 6000 V). Next, the induction heating coil was activated to heat the filler metal to 390 °C. After that, the heating was stopped and the capacitor was discharged to complete the brazing forming process, as shown in Figure 1c. The specific process parameters are listed in Table 1.

To clarify the formation mechanism of pores, the surfaces of the copper and aluminum tubes were treated. For the aluminum tubes, two surface treatments were applied: polishing with 3000-grit sandpaper and cleaning with chemical reagents. However, compared to sandpaper polishing, chemical reagent cleaning was more effective in removing oxide films and resulted in a smoother surface, which could reduce defects. The outer surface of the copper tubes had more oxides, oil stains, scratches, and impurities. Chemical cleaning could only remove oil stains and oxide films; additional polishing was required to meet the cleanliness and flatness requirements for brazing. Therefore, the copper tubes were polished with 100-grit, 3000-grit, and 5000-grit sandpapers separately.

### 2.3. Simulation Analysis

**Figure 2:**
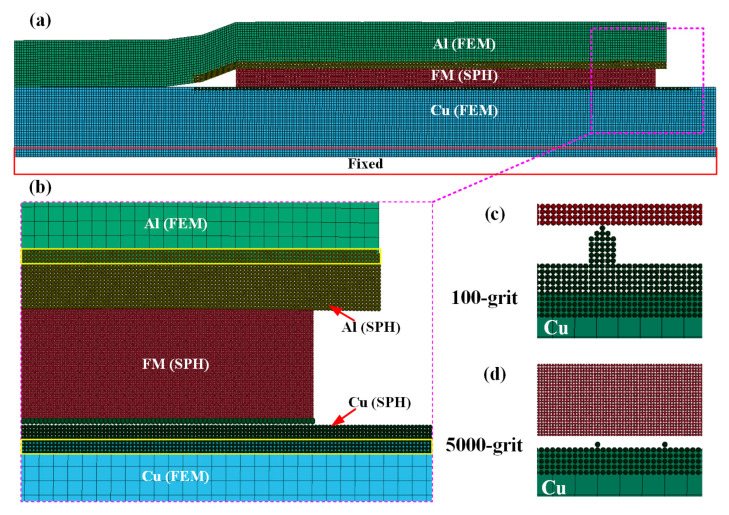
FEM-SPH model. (**a**) FEM-SPH model; (**b**) model details; (**c**) copper tube polished with 100-grit sandpaper; (**d**) copper tube polished with 5000-grit sandpaper.

In this study, LS-prepost 4.6 and LS-DYNA software were used for the keyword setting and subsequent simulation analysis of the FEM-SPH simulation. This FEM-SPH coupling method was used to analyze the pore formation mechanism during the solid–liquid interface interaction, and the corresponding modeling approach is shown in Figure 2. This model consists of two key components, Lagrangian meshes and SPH particles, and this combination is specifically designed to balance computational accuracy and efficiency. To be more specific, the Cu/Al tube interface and the filler metal region were constructed using SPH particles, as this approach allows for more accurate simulation of the dynamic interfacial interaction that occurs when the aluminum tube extrudes the filler metal. For all remaining regions, Lagrangian meshes were adopted. This choice not only effectively reduces the overall computational time but also makes it easier to apply load conditions consistent with the experiment in subsequent steps. Detailed procedures for model construction and viscosity parameters are available in the authors’ previously published research [11]. The physical property parameters of copper and aluminum can be referenced from the study by Xiong [13].

Additionally, to simulate how the copper tube surface roughness influences joint pore formation, the copper tube surface was specially treated during modeling. As shown in Figure 2c, the surface of the copper tube polished with 100-grit sandpaper exhibits distinct protruding structures. In contrast, Figure 2d presents the copper tube surface polished with 5000-grit sandpaper, which features a much smoother overall morphology and significantly reduced surface undulations.

## 3. Results

### 3.1. Analysis of Joint Macromorphology

**Figure 3:**
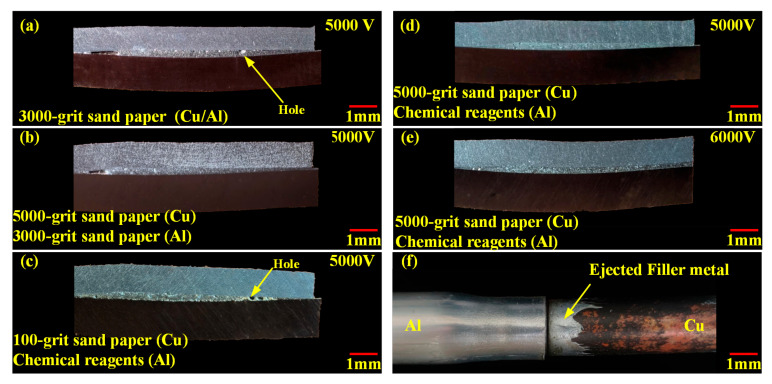
Macromorphology of joints obtained under different process parameters: (**a**) 5000 V/Al (3000-grit sandpaper)/Cu (3000-grit sandpaper); (**b**) 5000 V/Al (3000-grit sandpaper)/Cu (5000-grit sandpaper); (**c**) 5000 V/Al (chemical cleaning)/Cu (100-grit sandpaper); (**d**) 5000 V/Al (chemical cleaning)/Cu (5000-grit sandpaper); (**e**) 6000 V/Al (chemical cleaning)/Cu (5000-grit sandpaper); (**f**) Cu/Al tube joint.

Figure 3 presents the macromorphology of joints obtained under different process parameters. As can be seen from Figure 3f, after the experiment was completed, the filler metal was squeezed out by the aluminum tube. From Figure 3a,c, a key phenomenon is observed. There are many pores distributed in the brazing seam.

To analyze the effect of copper tube roughness, a comparison of Figure 3a,b is conducted. In both cases, the aluminum tubes were pre-polished with 3000-grit sandpaper, while the copper tubes used 3000-grit and 5000-grit sandpaper. The results show no obvious pores in the brazing seam when the copper tube was polished with 5000-grit sandpaper. A similar trend emerges from the comparison of Figure 3c,d. Here, the copper tubes were pre-polished with 100-grit and 5000-grit sandpaper. Large-sized pores appeared in the brazing seam for the 100-grit case, but no obvious pores were observed for the 5000-grit case. This confirms that the surface roughness of the copper tube is the main cause of pores in the brazing seam.

Further comparison of Figure 3d,e shows that when the copper tube was polished with 5000-grit sandpaper, no obvious pores were observed in the brazing seam even as the voltage is increased to 6000 V. In summary, fine polishing of the copper tube before brazing can effectively inhibit the formation of pores in the joint.

### 3.2. Analysis of Microstructure of Typical Joints

**Figure 4:**
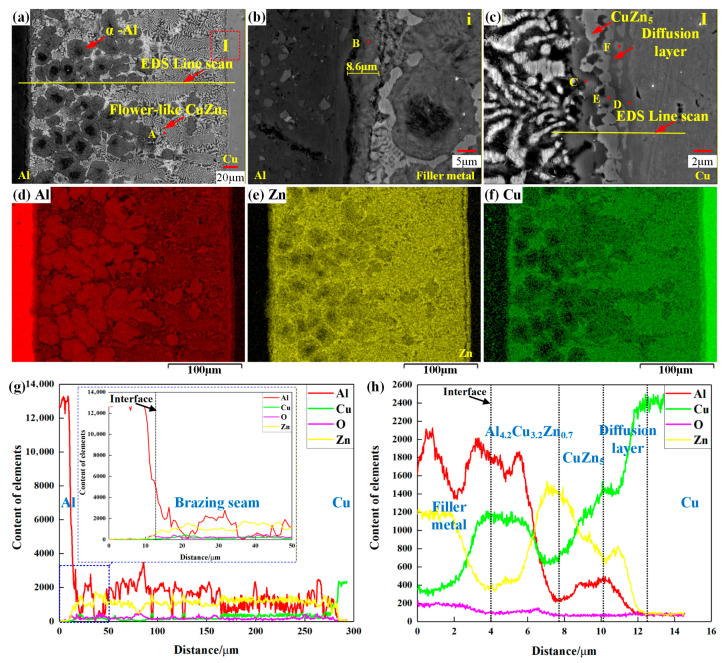
Microstructure and element distribution of the joint at brazing forming voltage of 5000 V and brazing temperature of 390 °C. (**a**) Cu/Al tube joint; (**b**) morphology of aluminum–side interface; (**c**) morphology of copper–side interface; (**d**–**f**) distribution of Al, Cu, and Zn elements; (**g**) element line analysis of (**a**); (**h**) element line analysis of copper–side interface.

Figure 4 presents the microstructure and element distribution at the top of the joint fabricated under a brazing forming voltage of 5000 V and a brazing temperature of 390 °C. As observed in Figure 4a, the brazing seam consists of α-Al and dendritic structures. EDS point analysis was performed on the dendritic structure (Point A in Table 2). Combined with the study by Paidar [3], this structure contains 66.8 at.% Zn, 15.2 at.% Cu, and 2.8 at.% Al, which is identified as the CuZn_5_ phase. The element distribution results in Figure 4d–f indicate that Zn, Al, and Cu elements can diffuse within the joint. Specifically, when Cu atoms diffuse into the brazing seam, they combine with Zn atoms to form dendritic CuZn_5_. The line scan analysis in Figure 4f further reveals a higher Cu concentration near the copper–side interface, which facilitates the formation of a large number of dendritic CuZn_5_ phases in this region.

Figure 4b shows the microstructure of the aluminum–side interface, where a diffusion layer with a width of 8.6 μm is formed. No brittle Cu/Al IMCs are detected. Consistent with the line scan results in Figure 4g, both Cu and Zn elements can diffuse toward the aluminum–side interface, but their contents are much lower than that of Al. Further, the EDS point analysis (Point B in Table 2) confirms that the Cu content in the diffusion layer is extremely low, only 0.75 at.%.

Figure 4c displays the microstructure of the copper–side interface, where metallurgical bonding is achieved, and two phases (dark gray and light gray) are observed. EDS point analysis of the dark gray phase (Point C in Table 2) shows a composition of 51.2 at.% Al, 29.4 at.% Cu, and 12.5 at.% Zn. Referring to the study by Zhuo [14], this phase is determined to be Al_4.2_Cu_3.7_Zn_0.7_. For the light gray phase (Point E in Table 2), EDS analysis yields a composition of 60.6 at.% Zn, 26.5 at.% Cu, and 12.0 at.% Al—this composition is close to that of the dendritic CuZn_5_ at Point A, so the light gray phase is also identified as CuZn_5_ according to the study by Liu [15]. Thus, under the conditions of 5000 V brazing forming voltage and 390 °C brazing temperature, the CuZn_5_ phase forms first at the copper–side interface, while the formation of Al_4.2_Cu_3.7_Zn_0.7_ depends on the diffusion degree of Al elements toward the copper–side interface. As shown in Figure 4h, the CuZn_5_ phase has the highest Zn content, followed by Cu, and the lowest Al content; in contrast, the Al_4_._2_Cu_3_._2_Zn_0_._7_ phase has the highest Al content, which is favorable for its formation.

In addition, EDS point analysis of Points D and F reveals a banded copper-based diffusion layer containing Cu_5_Zn_8_ [16] between the light gray CuZn_5_ phase and the Cu matrix. In summary, the Cu/Al tube joint fabricated by the MPASSB method achieves metallurgical bonding at low temperatures, and no brittle Cu/Al IMCs are formed at the joint interface.

### 3.3. Analysis of Pore Formation Mechanism Under Solid–Liquid Interface Interaction

#### 3.3.1. Analysis of Copper Tube Surface Roughness

**Figure 5:**
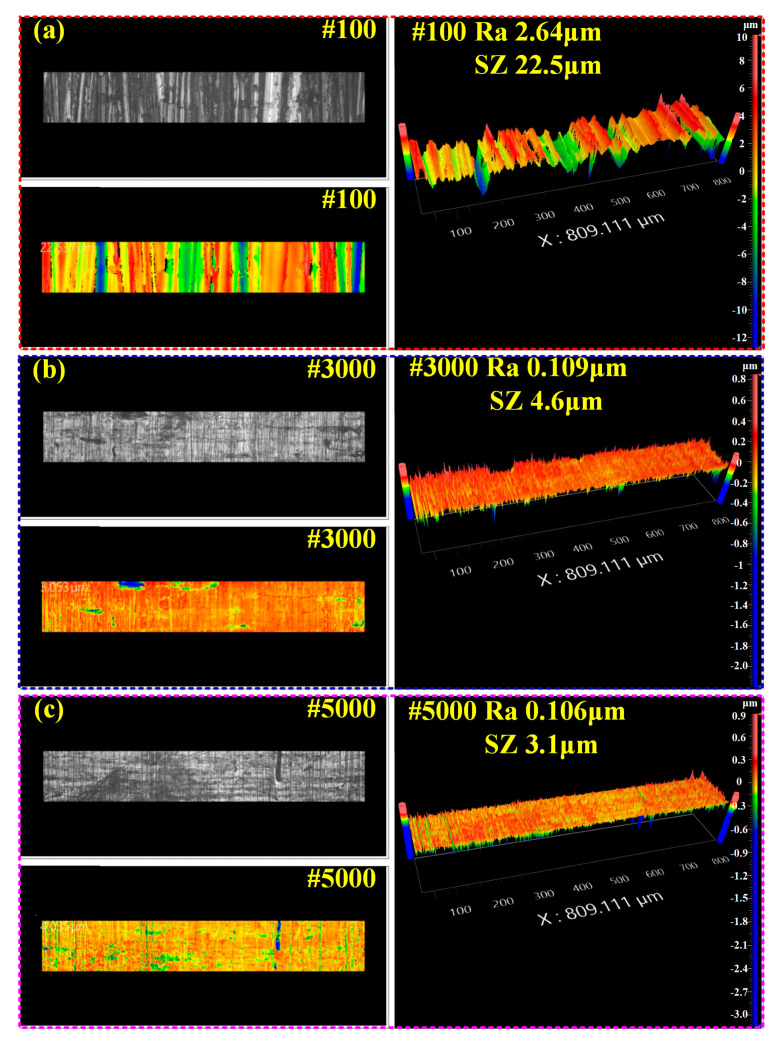
Surface roughness contour map of copper tube under different sandpaper polishing conditions. (**a**) 100-grit sandpaper; (**b**) 3000-grit sandpaper; (**c**) 5000-grit sandpaper.

Figure 5 shows the surface roughness contour maps of the copper tube under different sandpaper polishing conditions. Specifically, Figure 5a, Figure 5b, and Figure 5c correspond to the roughness contour maps of the copper tube polished with 100-grit, 3000-grit, and 5000-grit sandpaper, respectively. When combined with the data in Table 3, a clear trend emerges: as the grit size of the sandpaper increases, the surface roughness (Ra) of the polished copper tube decreases gradually. The specific Ra values for the three sandpaper grits are 2.64 μm (100-grit), 0.109 μm (3000-grit), and 0.106 μm (5000-grit).

In addition, Figure 5 also provides data on SZ, which represents the difference between the protrusions and depressions on the polished copper tube surface. For the 100-grit, 3000-grit, and 5000-grit sandpaper-polished surfaces, the SZ values are 22.5 μm, 4.6 μm, and 3.0 μm, respectively.

In summary, these results confirm a consistent pattern. As the grit size of the sandpaper increases, both the Ra and SZ values of the copper tube surface decrease gradually. This reduction in Ra and SZ also means that the protrusions on the copper tube surface weaken accordingly with the increase in sandpaper grit size.

#### 3.3.2. Effect of Copper Tube Surface Roughness on Joint Microstructure

**Figure 6:**
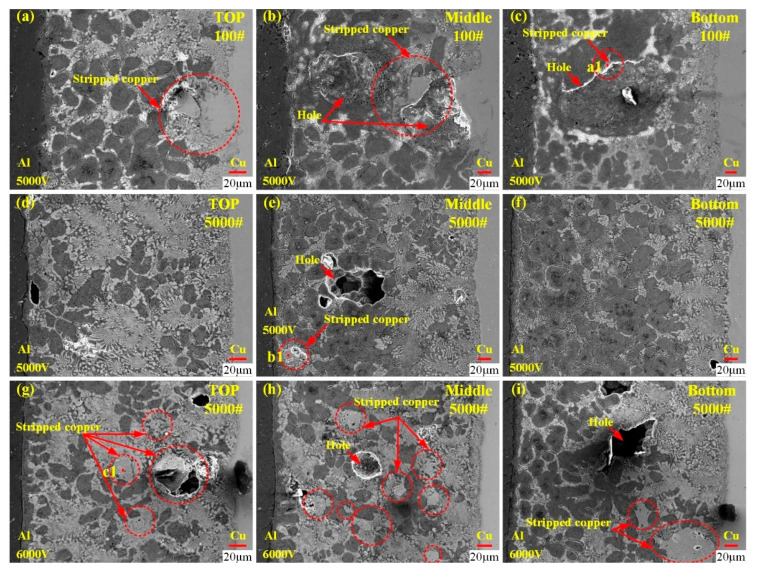
Joint microstructures obtained under different brazing forming voltages and copper tube surface roughness; (**a**–**c**) 5000 V/100-grit sandpaper; (**d**–**f**) 5000 V/5000-grit sandpaper; (**g**–**i**) 6000 V/5000-grit sandpaper.

Figure 6 shows the joint microstructures obtained under different brazing forming voltages and copper tube surface roughness. It should be noted that in all the experiments corresponding to Figure 6, the aluminum tube surfaces were only cleaned with chemical reagents, with no sandpaper polishing applied. Specifically, Figure 6a–c present the joint microstructures obtained at a brazing forming voltage of 5000 V, where the copper tube was polished with 100-grit sandpaper; Figure 6d–f also correspond to a brazing forming voltage of 5000 V, but with the copper tube polished with 5000-grit sandpaper; and Figure 6g–i show the joint microstructures obtained at a higher brazing forming voltage of 6000 V, with the copper tube still polished with 5000-grit sandpaper.

First, comparing Figure 6a,d,g reveals a key trend. As the sandpaper grit size for copper tube polishing increases from 100 to 5000, the flatness of the copper–side interface improves significantly. This observation aligns with the surface roughness analysis results presented in Figure 5, further confirming that the higher-grit sandpaper effectively refines the copper tube surface. Further analysis of the 100-grit sandpaper case (see Figure 6a–c) uncovers distinct defects. Large-sized pores appear in the middle and bottom of the joint (as seen in Figure 6b,c), and there are obvious copper block residues at these pore sites (see Point a1 in Table 4). Additionally, in Figure 6a, the copper blocks protruding from the copper–side interface at the top of the joint show a tendency to move toward the brazing seam during their interaction with the filler metal. This movement may potentially contribute to pore formation.

In contrast, for the 5000-grit sandpaper case (see Figure 6d–i), small copper blocks appear in the brazing seam regardless of whether the brazing voltage is 5000 V or 6000 V (see Points b1 and c1 in Table 4). However, a notable difference emerges with changes in voltage. When the voltage is increased to 6000 V, the number of both small copper blocks and small pores in the brazing seam becomes significantly higher than that observed at 5000 V. This observation indicates that higher voltage may exacerbate minor defect formation even with a smooth copper tube surface.

In summary, two main conclusions can be drawn from Figure 6. Refining the tube surface roughness, that is specifically using higher-grit sandpaper for copper tube polishing, helps reduce the size and number of pores in the joint. Meanwhile, under the premise of meeting the joint service requirements, the brazing forming voltage should be minimized as much as possible to avoid excessive pore formation. However, it should be noted that based on the current experimental results, the effect of the interaction between the filler metal and the tubes on pore formation cannot be clearly determined; the specific relationship between these two factors thus remains unclear. This relationship therefore requires further in-depth analysis in subsequent studies.

#### 3.3.3. FEM-SPH Simulation

**Figure 7:**
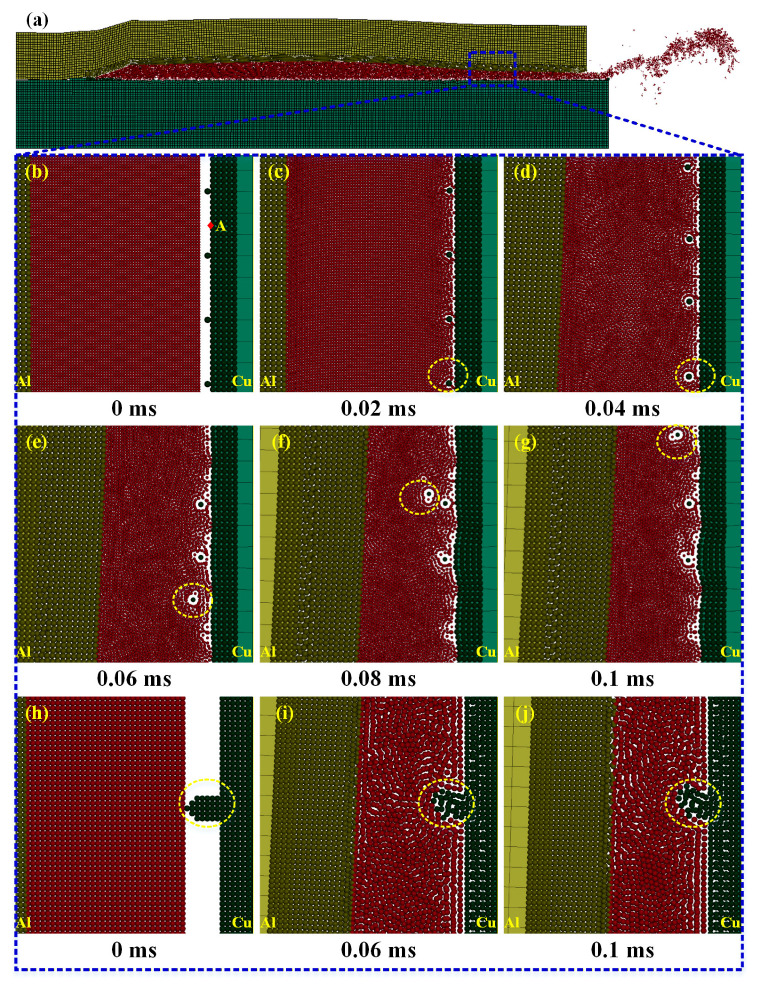
FEM-SPH simulation analysis of pore formation. (**a**) FEM-SPH results; (**b**–**g**) copper tube polished with 5000-grit sandpaper, brazing forming voltage of 6000 V; (**h**–**j**) copper tube polished with 100-grit sandpaper, brazing forming voltage of 5000 V.

**Figure 8:**
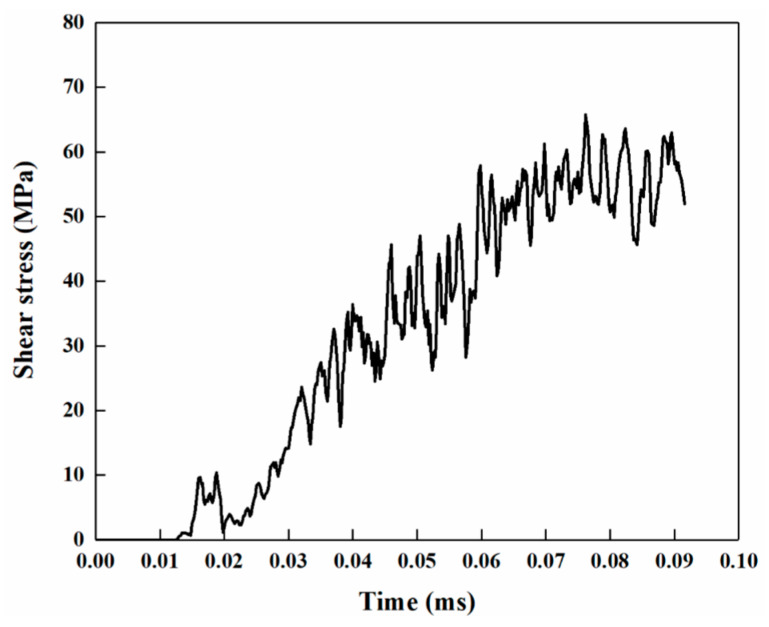
Curve of shear stress at the copper–side interface versus time.

Figure 7 presents the FEM-SPH simulation results of joints with different surface roughness of copper tubes. Figure 8 shows the curve of shear stress at the copper–side interface versus time, where the test region corresponds to Point A in Figure 7b. As indicated in Figure 7a, the FEM-SPH simulation can more realistically reproduce the phenomenon of filler metal ejection after high-speed impact.

In Figure 7b–g and Figure 7h–j, the copper tubes were polished with 5000-grit and 100-grit sandpapers, respectively. For the copper tube polished with 5000-grit sandpaper, the copper–side interface is smooth, with small, raised copper particles distributed intermittently. In contrast, the copper tube polished with 100-grit sandpaper exhibits large-sized raised copper blocks.

From Figure 7b–j and Figure 8, when the aluminum tube extrudes the filler metal, the filler metal undergoes high-speed shear flow and interacts with the copper tube surface. The interfacial shear stress gradually increases during the brazing forming process, reaching a peak of 65.7 MPa at 0.065 ms. Under the 5000-grit sandpaper polishing condition, the copper particles (marked by yellow circles) are small in size and have weak bonding strength with the copper matrix; these particles are detached from the copper tube surface under the action of shear stress. For the 100-grit sandpaper polishing condition, the raised copper blocks are large in size and have high bonding strength with the copper matrix. The interface shear stress cannot detach them from the copper matrix, but only tears the top of the copper blocks, which are likely to flow into the brazing seam with the filler metal and form pores.

Further combined with Figure 6, when the surface roughness of the copper tube is low, only the small copper particles appear in the brazing seam. These small particles are more likely to mix tightly with the brazing seam, resulting in a low probability of pore formation and minimal impact on joint performance and air tightness. In contrast, high surface roughness causes large-sized copper blocks to detach from the copper–side interface and enter the brazing seam. These large blocks are difficult to mix tightly with the brazing seam, leading to a higher probability of defect formation.

Therefore, to avoid such defects, the forming voltage should be minimized under the premise of effective oxide film removal, preventing excessive interaction at the solid–liquid interface. Meanwhile, the surface roughness of Cu/Al tubes should be reduced, and specifically, aluminum tubes should be cleaned with chemical reagents while copper tubes should be polished with 5000-grit or finer sandpapers.

### 3.4. Analysis of Joint Microstructure Evolution Mechanism

As observed in Figure 4a–c, the oxide layer at the joint interface is completely removed during the Cu/Al tube joining process using the MPASSB method, and this complete removal serves as a prerequisite for achieving interfacial metallurgical bonding. The mechanism behind the oxide layer removal lies in the brazing forming stage. First, the high-speed shear flow of the filler metal generates shear stress at the copper–side interface (see Figure 8). Then, driven by the continuous flow of the filler metal, the oxide layer is peeled off from the copper–side interface and further fragmented [11].

**Figure 9:**
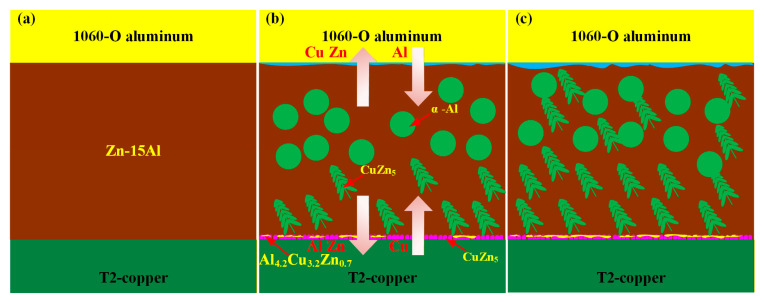
Schematic diagram of element diffusion and microstructure evolution in MPASSB Cu/Al tube joint. (**a**) Initial state; (**b**) schematic of element diffusion; (**c**) schematic of microstructure after brazing formation.

Figure 9 presents a schematic diagram of element diffusion and microstructure evolution in the MPASSB Cu/Al tube joint. As brazing forming proceeds, at the aluminum–side interface, a large amount of Al dissolves into the filler metal, while Zn diffuses into the Al matrix through the grain boundaries. This process is the key to the formation of the diffusion layer. In the brazing seam, the diffusion of Cu into the seam promotes the growth of dendritic CuZn_5_ phases along the copper–side interface. At the copper–side interface, Zn exhibits a higher diffusion rate into the copper matrix compared to Al [17], and this difference in diffusion rates is the key factor for the preferential formation of CuZn_5_ phases at the interface.

As the brazing proceeds further, the diffusion of Al towards the copper matrix and the diffusion of Cu towards the brazing seam both become enhanced. This enhanced element diffusion drives the dendritic CuZn_5_ phases to grow throughout the entire brazing seam. Meanwhile, the Al_4.2_Cu_3.7_Zn_0.7_ phase forms at the outer side of the CuZn_5_ phases, completing the full evolution of the joint microstructure.

### 3.5. Mechanical Properties of Joints

**Figure 10:**
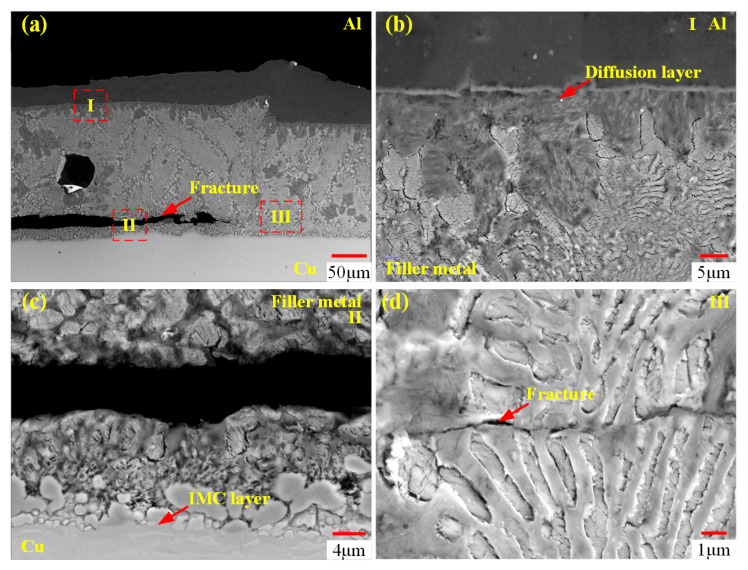
Fracture analysis of the joint at a brazing forming voltage of 5000 V. (**a**) Joint at low magnification; (**b**) magnified view of region I (aluminum–side interface) in (**a**); (**c**) magnified view of region II (copper–side interface) in (**a**); (**d**) magnified view of region III (brazing seam) in (**a**).

Based on the above analysis, the optimal process parameters are determined as follows: a brazing forming voltage of 5000 V, aluminum tubes cleaned with chemical reagents, and copper tubes polished with 5000-grit sandpaper. Figure 10 shows the fracture morphology of the joint under these optimal parameters. Figure 10a shows that the joint fracture occurs at the copper–side interface. To further examine interface characteristics, Figure 10b provides a magnified view of region I at the aluminum–side interface of Figure 10a. Meanwhile, Figure 10c and Figure 10d offer magnified views of region II and region III at the copper–side interface, respectively.

From Figure 10b, no fracture is observed at the aluminum–side interface. From Figure 10c,d, it can be seen that fracture occurs at the brazing seam near the copper–side interface, but no fracture occurs at the IMC layer. Mechanical property tests show that the joint shear strength reaches 63.3 MPa.

A comparison to existing research results on Cu/Al joints shows the following characteristics. Niu [18] used muffle furnace brazing with Zn-15Al filler metal to join copper and aluminum, and the resulting joint exhibited a shear strength of 70–75 MPa. This strength, however, is significantly affected by brazing temperature and time; it decreases to 45 MPa under high temperatures and long brazing durations due to the massive formation of the Al_2_Cu phase. Xiao [19] adopted ultrasonic-assisted brazing with Zn-3Al filler metal for Cu/Al joining, and the joint shear strength was most notably influenced by brazing temperature, reaching a maximum value of 78.9 MPa at 440 °C. Neither of these two methods, however, can achieve effective joining of copper and aluminum tubes. Flame brazing is currently the most commonly used method for Cu/Al tube joining [20], but joints prepared by this method have a shear strength of only approximately 54 MPa due to the extensive formation of the Al_2_Cu phase.

In contrast, the Cu/Al tube joint, prepared by the MPASSB method, not only has higher strength than that of flame-brazed joints but avoids the formation of brittle Cu/Al phases as well. In summary, the MPASSB method exhibits high application value and potential in the field of Cu/Al joint preparation, and is expected to provide more high-quality joining solutions for related engineering fields.

## 4. Conclusions

(1) The MPASSB method enables the fabrication of Cu/Al tube joints with excellent metallurgical bonding at a low brazing temperature (390 °C). A diffusion layer is formed at the aluminum–side interface of the joint, while the copper–side interface consists of CuZn_5_ and Al_4.2_Cu_3.7_Zn_0.7_ phases. No brittle IMCs are detected in the joint.
(2) The joints exhibit good mechanical properties, with a shear strength of 63.3 MPa and fractures occurring in the brazing seam area adjacent to the copper–side interface.
(3) The FEM-SPH approach is well-suited for the simulation of solid–liquid coupling interactions. The solid–liquid interface interaction and the surface roughness of the tubes are the key factors influencing pore formation in the joint. Under the premise of ensuring effective joint bonding, the brazing forming voltage and the surface roughness of the tubes should be minimized as much as possible.

## Figures and Tables

**Table 1:** Experimental scheme.

	Clamping Forming Voltage (V)	Brazing Forming Voltage (V)	Surface Cleaning (Sandpaper)	
			Cu	Al
1	5000	5000	3000-grit	3000-grit
2	5000	5000	5000-grit	3000-grit
3	5000	5000	100-grit	chemical reagents
4	5000	5000	5000-grit	chemical reagents
5	5000	6000	5000-grit	chemical reagents

**Table 2:** EDS analysis results of the points marked in Figure 4.

Point	At%				Possible Phase
	Al	Cu	Zn	O	
A	2.8	15.2	66.8	15.1	CuZn_5_
B	68.45	0.75	19.3	11.5	Diffusion layer
C	51.2	29.4	12.5	6.9	Al_4.2_Cu_3.2_Zn_0.7_
D	19.3	48.5	27.6	4.6	Diffusion layer
E	12.0	26.5	60.6	0.9	CuZn_5_
F	9.6	41.1	47.1	2.3	Cu_5_Zn_8_

**Table 3:** Surface roughness of the tube polished with different sandpapers in Figure 5.

	Ra μm	SZ μm
100-grit	0.045	22.5
3000-grit	0.78	4.6
5000-grit	2.22	3.1

**Table 4:** EDS point analysis in Figure 6.

Analysis Point	At.%				Possible Phase
	Al	Cu	Zn	O	
a1	0.3	90.2	6.6	2.9	Cu
b1	13.1	70.8	15.8	0.3	Cu
c1	7.8	82.3	6.7	3.2	Cu

## Data Availability

The original contributions presented in this study are included in the article. Further inquiries can be directed to the corresponding author.
